# Coconut husk-raw clay-Fe composite: preparation, characteristics and mechanisms of Congo red adsorption

**DOI:** 10.1038/s41598-022-18763-y

**Published:** 2022-08-23

**Authors:** Matthew Ayorinde Adebayo, Jamiu Mosebolatan Jabar, Justinah Solayide Amoko, Elijah Ojo Openiyi, Olamide Oladimeji Shodiya

**Affiliations:** 1 Department of Chemistry, The Federal University of Technology, Akure, Ondo State Nigeria; 2 Department of Chemistry, Adeyemi College of Education, Ondo, Ondo State Nigeria; 3 Interdisciplinary Ecological Sciences and Engineering, Purdue University, West Lafayette, IN 47906 USA

**Keywords:** Environmental sciences, Chemistry, Materials science

## Abstract

The release of unspent dyes from industries constitutes hazard and environmental challenges. For rapid and efficient removal of Congo red from aqueous solutions, a composite was prepared from coconut husk, raw clay, Fe(II) and Fe(II) compounds. Adsorption variables (initial pH of the solution, contact time, temperature and initial concentration of Congo red) were varied to understand the characteristics and mechanisms of the adsorption process. The composite was characterised using Fourier Transform Infrared (FTIR) spectroscopy, Scanning Electron Microscopy (SEM)–Energy dispersive X-ray (EDX) spectroscopy, nitrogen adsorption–desorption isotherm, X-ray Diffraction (XRD) spectroscopy and pH of the point zero charge (pH_pzc_). The optimal values of the pH, equilibrium time and temperature for adsorption of Congo red by the composite are 2, 40 min and 50 °C, respectively. The kinetic and equilibrium data followed Avrami fractional order and Langmuir models, respectively. A 1.0 g of the composite could maximally take up 1649.3 mg of Congo red at 50 °C. The values of Δ*G*° are in the range of − 27.901 to − 24.492 kJ mol^–1^ while the value of Δ*H*° is − 72.239 kJ mol^–1^. Hence, the removal of the Congo red by the composite was spontaneous, feasible and exothermic. The adsorption process was biphasic and followed physisorption process. Electrostatic interaction played a significant role in the removal of Congo red by the composite. The combine data in this study have proven that the clay composite, a cheap adsorbent, can be used for remediation of water contaminated with Congo red.

## Introduction

Clean water is an essential commodity needed by man for existence, sustenance of life and good health. Most water sources, however, are continuously being polluted. Water pollution is regarded as one of the environmental challenges nowadays because water pollution affects ecological and economic systems globally^1^. Varieties of dyes are useful industrially for formulation or production of food, paper and pulp, ink, printing, leather, plastic, textiles, soap and cosmetics, among others^2^. The release of unspent dyes, in the form of coloured wastewater, is disastrous because dye molecules in water can result in cancer, toxicity, allergic reactions, gene mutation, respiratory and lungs diseases, and dermatitis in human beings as well as reduction of sunlight penetration in water, which could lead to a decrease in photosynthetic rate in aquatic plants^2,3^. Different categories of dye-contaminated wastewater are released, without treatment, into natural water and environment.

Congo red, a benzidine-derived anionic disodium salt or diazo dye, is popularly employed for dyeing of textile materials due to its chromaticity. This dye has complex chemical structure and it is known to form carcinogenic product(s) when metabolised to benzidine and can induce allergic reactions^4^. Congo red has high water solubility and changes colour in aqueous solutions depending on the pH of the solutions, and hence the occurrence of Congo red in water gives rise to offensive changes in the colour of water, and this poses a danger to aquatic flora^5^. It is also widely known that Congo red exists in different molecular forms in aqueous solutions depending on the pH and this characteristic makes it difficult for outright elimination of Congo red in solution^6^.

Dyes are mostly non-biodegradable and can be removed from water systems through biological, chemical or physicochemical treatment method or combination of the methods^7^. Adsorptive elimination of recalcitrant dye molecules from contaminated water and wastewater is considered most effective and efficient because most of the adsorbent materials are low-cost, green and eco-friendly, easily available, renewable, and efficient^8^. Different materials that have been successfully employed for adsorption of Congo red from aqueous solutions include amino-functionalised silica gel^9^, activated carbon coffee waste^10^, bark of Pine^7^, clay–corn cob–FeCl_3_^11^, Periwinkle shell activated carbon^12^, Ackee apple seed–bentonite composite^3^, modified rice husk char^13^, synthesised coal graphene^5^ and Water hyacinth bark carbon^14^.

Cheap adsorbent materials can be obtained from agricultural wastes, household wastes, sludge, by-products from industrial processes, soil (e.g. clay), ores, aquatic materials, and other newly developed adsorbents^15^. Agricultural wastes and lignocellulosic materials are cheap, readily available and effective materials for production of adsorbents^10^. Agricultural wastes, which are rich in lignin, cellulose, and hemicellulose, possess functional groups such as hydroxyl, amino, carboxyl, carboxyl, and methyl that can adsorb dye molecules from aqueous solutions^16^. Coconut husk is an agricultural by-product, which is discarded indiscriminately at times. Coconut husk is a fluffy coconut material that is produced during the separation process of the coconut fibre from the coconut husk^17^. The discard and accumulation of this by-product can lead to serious environmental menace because microbial degradation of the waste can lead to production of toxic substances. In most countries, the husks are burnt to prevent accumulation and this act of burning is contributing to CH_4_ and CO_2_ emission. About 8,000,000 tonnes of coconut husk is being generated yearly.

On the basis of the fascinating features (such as cheapness, high surface areas, ion exchange capacity, high-binding potentials for pollutants, availability, abundance, and non-toxicity) of clay and clay-related materials, they are effectively, technically and economically suitable materials for adsorption of diverse pollutants from wastewater^15,18^. To enhance performance of clay materials for dye removal, surface modifications of these materials are required. Surface modification of clay materials can be done via chemical treatment (e.g. acidification, addition of quaternary alkylammonium ions) or physical treatment (e.g. calcination) or combination of both methods^19^.

A number of studies have examined adsorption of Congo red from solutions using clay materials. Vahidhabanu et al.^20^ synthesised ZnO-clay-alginate beads for Congo red removal using batch and packed column systems. In their report, the adsorbent possessed surface and functional groups that are suitable for removal of Congo red and the adsorbent could favourably remove 546.89 mg g^–1^ of the dye in a batch mode. In another report by Olusegun and Mohallem^21^, Congo red was removed effectively from aqueous solution using kaolinite-CoFe_2_O_4_ in a batch adsorption process. A 1 g of the synthesised composite could remove 547 mg of Congo red from water at 298 K. Bentonite-1-dodecyl-3-methylimidazolium chloride composite was reported for Congo red removal from aqueous solutions by Ozola-Davidane et al.^22^. The eco-friendly modified bentonite has adsorption capacity of 150 mg g^–1^ for Congo red in aqueous solution. Abou Alsoaud et al.^23^ reported synthesis of kaolin-aminated-chitosan composite for adsorption of Congo red dye from water. The adsorbent had the capacity to remove 104.16 mg of the dye from aqueous solution at pH 7. Despite the successes recorded by these researchers and others in getting rid of Congo red from water, efforts are continuously been made to synthesise effective adsorbents for water and wastewater treatment.

In a search for alternative, inexpensive and effective adsorbent material for sequestration of Congo red from water, a composite was prepared from clay, coconut husk, and iron salts. The iron salts, termed magnetic compounds, were included in the preparation of the adsorbent to improve the binding, porosity and adsorption properties of the composite. Combining outstanding adsorptive properties of clay with functional groups present in coconut husk (biomass), will result in the formulation of an excellent adsorbent. In the synthesis of the composite, cheapness and availability of the precursors (clay and coconut husk), utilisation of green chemicals, non-consumption of energy, and non-complicated synthetic method were considered. Adsorption characteristics of the composite were probed by varying the pH of the solution, contact time, concentration of Congo red, and experimental temperature. Possible mechanisms of adsorption process of the composite are proposed.

## Materials and methods

### Preparation of the composite

Analytical grades of reagents were used without further treatment. Congo red (CAS No., 573-58-0; molecular formula, C_32_H_22_N_6_Na_2_O_6_S_2_; percentage purity, 97%; molecular weight, 696.66 g mol^–1^) was furnished by Sigma-Aldrich, Germany. The chemical structure of the dye is shown in Supplementary Fig. S1 of the Supplementary Information. Iron (II) sulphate (CAS No., 7782-63-0; molecular formula, FeSO_4_·7H_2_O; percentage purity, 97.5%; molecular weight, 278.01 g mol^–1^), iron (III) sulphate (CAS No., 15244-10-7; molecular formula, Fe_2_(SO_4_)_3_·H_2_O; percentage purity, 73%; molecular weight, 417.87 g mol^–1^), calcium oxide (CAS No., 1305-78-8; molecular formula, CaO; percentage purity, 99.99%; molecular weight, 56.08 g mol^–1^), calcium hydroxide (molecular formula, Ca(OH)_2_; percentage purity, 95%; molecular weight, 74.093 g mol^–1^), and calcium carbonate (CAS No., 471-34-1; molecular formula, CaCO_3_; percentage purity, 97.5%; molecular weight, 100.09 g mol^–1^) were supplied by Thermo Scientific. Distilled water was used to prepare all the solutions.

Coconut husks were obtained from a dumping site of a local market in Akure, Nigeria while raw clay was furnished by the Department of Industrial Design, The Federal University of Technology, Akure, Nigeria. The two precursory materials were taken to the Department of Crop, Soil and Pest Management of The Federal University of Technology, Akure, Nigeria for proper identification and authentication. The coconut husks were washed with tap water and subsequently with distilled water, sun-dried for 7 days to drive off the moisture, pulverised and sieved with a 2 mm mesh sieve to get a sample of smooth and small particles. The sample was properly kept until usage. The raw clay sample was sun-dried for 5 days, oven-dried at 90 °C for 24 h, crushed with mortar and pestle, sieved with a 2 mm mesh to get clay of fine particles, and kept until usage.

A 100 g of the husk, 100 g of the raw clay, and 300 mL of distilled water were adequately mixed and this mixture was labelled as mixture A. In another preparation, a 40 g ferric sulphate (Fe_2_(SO_4_)_3∙_H_2_O) was weighed and dissolved in 50 mL of water while 40 g of ferrous sulphate (FeSO_4_·7H_2_O) was also weighed and dissolved in 50 mL of water. The two solutions were mixed together, and this formed mixture B. Lime (20 g) was prepared by weighing and mixing CaO, Ca(OH)_2_ and CaCO_3_, and dissolving in 50 ml of water and this solution was named as mixture C. The three mixtures were mixed and stirred on the magnetic stirrer for 1 h at 70 °C. To the resulting mixture still on the magnetic stirrer, a few drops of 10 mol L^–1^ NaOH was added to the solution while stirring to maintain a pH of 10^24^. The final mixture was allowed to stand for 24 h for proper equilibration. The clear solution was removed and the residue (adsorbent) was washed with distilled water, oven-dried for 48 h at 80 °C, sieved with a 106 µm mesh sieve, labelled as CHCFe (coconut husk-clay-ferrate composite), and kept in an airtight plastic container in a desiccator until usage. The lime in the composition was to allow proper mixing of the components^25^.

### Techniques for characterisation of the composite

The functional groups of the composite were obtained using Fourier Transform Infrared Spectroscopy (FTIR; Shimadzu Spectrometer, IR Prestige 21, Japan). The CHCFe and KBr were separately oven-dried at 393 K for 7 h, and stored in a desiccator before analysis. The spectrum of the CHCFe was recorded between 4000 and 400 cm^–1^. The surface feature of CHCFe was visualised using Scanning Electron Microscopy (SEM; Zeiss DSM 982 Gemini). The specific surface area, pore size distribution and total pore volumes of CHCFe were obtained by using BET (Brunauer, Emmett and Teller) multipoint technique, and BJH (Barret, Joyner and Halenda) from N_2_ adsorption–desorption isotherm at 77 K using a surface analyser (Micrometrics TriStar II 3020). The composite was also subjected to X-ray diffraction (XRD) spectroscopy to obtain its diffraction patterns using Philips X’pert MPD diffractometer (Netherlands), which was operated at 40 kV and 40 mA with CuKa radiation (λ = 1.5406 Å). By using pH drift method^2,5,25^, the value of pH of the point zero charge (*pHpzc*) of CHCFe was obtained.

### Adsorption experiments, modelling and statistical analysis

A 5.00 g L^–1^ stock solution of Congo red was prepared and this solution was diluted serially for preparation of various working solutions. Removal of Congo red, using the composite, was achieved by using batch adsorption method. In various adsorption experiments to probe the effects of pH, contact time, initial concentration of Congo red and temperature on adsorption characteristics of CHCFe, 20 mL Congo red solutions (0.02–3.00 g L^−1^) was added to 0.05 ± 0.003 g of CHCFe in 25 mL Falcon tubes at varying pH values (2–10), contact times (0–480 min), and temperatures (25, 30, 35, 40 and 50 °C). Different dye solution-composite mixtures were agitated thermostatically on a shaker at 160 rpm (revolution per minute) and centrifuged at the end of the adsorption experiments for separation of adsorbent from un-adsorbed Congo red in solution. The concentration of the dye left (un-adsorbed) in the filtrate solution was measured at 497 nm on UV/Visible spectrophotometer (Shimadzu, Japan). The absorbance readings from the spectrophotometer were converted to concentrations using a standard calibration curve. All adsorption experiments were carried out three times, and the mean values were used for further analysis. The amount of Congo red adsorbed (*Q*_*e*_, mg g^−1^) from solution and the percentage of adsorption (%Ads) were evaluated with the aids of Eqs. (1) and (2), respectively.

1 $$Q_{e} = \frac{{\left( {C_{o} - C_{e} } \right) \times V}}{m}$$

2 $$\% Ads = \frac{{C_{o} - C_{e} }}{{C_{o} }} \times 100$$

where *C*_*o*_ and *C*_*e*_ stand for respective initial and final (equilibrium) concentrations of Congo red in mg L^−1^; *V* denotes the volume, in litre (L), of the aqueous phase; and *m* is the weight (g) of the CHCFe used for each experiment.

The data obtained from time-dependent experiment were subjected to non-linear forms of pseudo-first order, pseudo-second order, Modified Ritchie second order, Avrami fractional order and intraparticle diffusion kinetic models, which are shown respectively in Eqs. (3)–(7). The concentration and temperature-dependent (isothermal) data were further analysed using non-linear forms of Langmuir, Freundlich, Dubinin–Radushkevich (D–R), and Toth equilibrium models, which are presented in Eqs. (8)–(11), respectively.

3 $$Q_{t} = Q_{e} \left\{ {1 - \exp ( - k_{f} t)} \right\}$$

4 $$Q_{t} = Q_{e} - \frac{{Q_{e} }}{{k_{s} Q_{e} t + 1}}$$

5 $$Q_{t} = Q_{e} \left\{ {1 - \frac{1}{{k_{mR} t + \beta }}} \right\}$$

6 $$Q_{t} = Q_{e} \left\{ {1 - \exp ( - k_{Av} t)^{{n_{Av} }} } \right\}$$

7 $$Q_{t} = k_{ipd} \sqrt t + C$$

8 $$Q_{e} = \frac{{Q_{\max } K_{L} C_{e} }}{{1 + K_{L} C_{e} }}$$

9 $$Q_{e} = K_{F} C_{e}^{{\tfrac{1}{{n_{F} }}}}$$

10 $$Q_{e} = Q_{\max } \exp \left\{ { - K_{DR} RT\ln \left[ {1 + \frac{1}{{C_{e} }}} \right]^{2} } \right\}$$

11 $$Q_{e} = \frac{{Q_{\max } \cdot K_{T} \cdot C_{e} }}{{\left\{ {1 + \left( {K_{T} \cdot C_{e} } \right)^{{\frac{1}{{n_{T} }}}} } \right\}^{{n_{T} }} }}$$

In the above equations, *Q*_*t*_ is the amount (mg) of Congo red removed by 1.0 g of CHCFe at time, *t; k*_*f*_ denotes the pseudo-first order rate constant (min^–1^); *k*_*s*_ represents the pseudo-second order rate constant (g mg^–1^ min^–1^); *k*_*mR*_ stands for the modified Ritchie second order rate constant (min^–1^); β stands for the Ritchie’s constant, which is related to initial loading of the pollutant on adsorbent (min^–1^); *k*_*Av*_ represents the Avrami fractional order rate constant (min^–1^); *n*_*AV*_ denotes the Avrami’s fractional order, which is related to the mechanism of adsorption; *k*_*ipd*_ is the intraparticle diffusion rate constant (mg g^–1^ min^–0.5^); *C* represents the intraparticle diffusion constant, which is related to the thickness of the boundary layer (mg g^–1^); *Q*_*max*_ is the maximum adsorption capacity of the adsorbent (mg g^–1^); *C*_*e*_ represents the equilibrium concentration of the dye (mg L^–1^); *K*_*L*_ is the Langmuir equilibrium constant (L mg^–1^); *K*_*F*_ is the Freundlich equilibrium constant $(mg/g)(mg/L)^{{ - \frac{1}{{n_{F} }}}}$; *n*_*F*_ denotes the Freundlich’s exponent; *K*_*DR*_ is the activity coefficient, which is related to mean free energy of adsorption (mol^2^ kJ^−2^); *K*_*T*_ denotes the Toth equilibrium constant (L mg^−1^); and *n*_*T*_ stands for the Toth exponent (dimensionless).

Thermodynamic equations (Eqs. 12 and 13) were used to calculate the values of the standard Gibbs free energy change (*ΔG°*; kJ mol^–1^), standard enthalpy change (*ΔG°*; kJ mol^–1^) and standard entropy change (*ΔG°*; J mol^–1^ K^–1^).

12 $$\Delta G^{o} = - RT\ln K_{L}$$

13 $$\ln K_{L} = - \frac{{\Delta H^{o} }}{RT} + \frac{{\Delta S^{o} }}{R}$$

where *R* is the universal gas constant (8.314 J mol^–1^ K^–1^) and T denotes the temperature in K.

Non-linear curve fittings and calculations of parameters were done on OriginPro 9. The $R_{adj}^{2}$(adjusted determination coefficient) and *SD* (standard deviation) values of each model were used to determine the best model of the adsorption data. The statistical representations of $R_{adj}^{2}$ and *SD* are presented in Eqs. (14) and (15), respectively.

14 $$R_{adj}^{2} = \left\{ {1 - (1 - R^{2} )} \right\}\left\{ {\frac{n - 1}{{n - p - 1}}} \right\}$$

15 $$SD = \sqrt {\left\{ {\frac{1}{n - p}} \right\}\sum\limits_{i}^{n} {\left( {q_{i,\exp } - q_{i,\text{model}} } \right)^{2} } }$$

where *q*_*i,exp*_ represents an individual, *i*, data obtained from the batch experiment, *q*_*i,model*_ represents the estimation of the corresponding *q*_*i,exp*_ generated by each model; *n* is the number of data points; and *p* is the number of parameters in the model. A small value of *SD* indicates a good curve fit by the model while a value of $R_{adj}^{2}$ close to unity also signifies a good curve fit by a model^2,5^.

## Results and discussion

### Preparation and characterisation of CHCFe

In a quest for an effective adsorbent material for wastewater treatment, a composite (CHCFe) was prepared from coconut husk (an agricultural by-product), raw clay (abundant and cheap), magnetic particles (have a good tendency for capturing environmental pollutants^26^ and lime that ensured proper mixing of all the components^3,25^. The composite was successfully used for removal of Congo red from water. The characteristics of the adsorbent were elucidated by using SEM–EDX, FTIR, XRD, nitrogen adsorption–desorption, and *pH*_*pzc*_.

**Figure 1:**
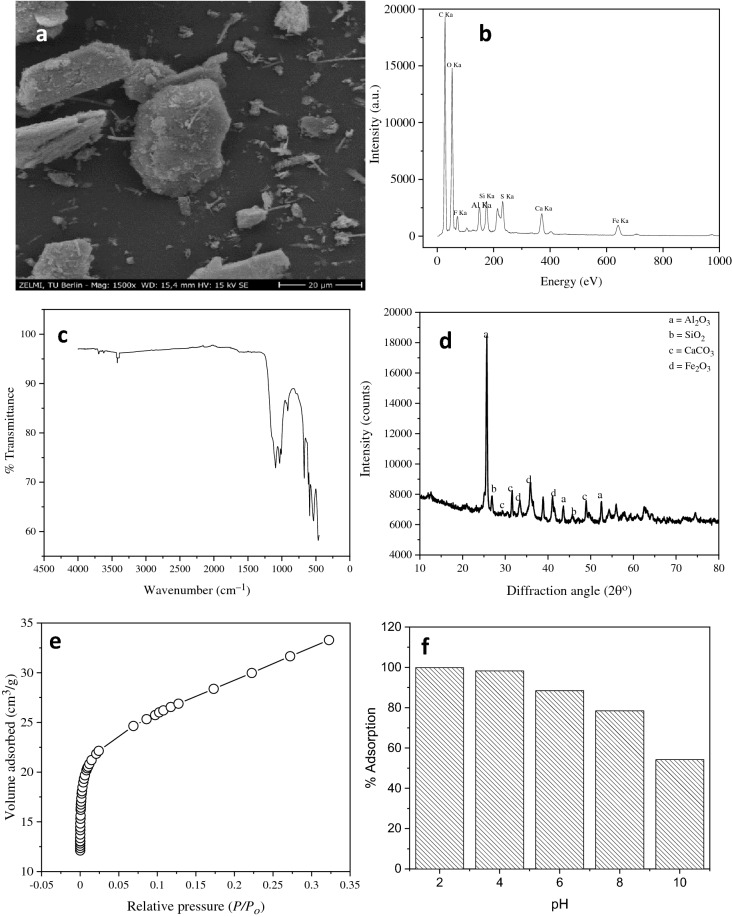
Characteristics of CHCFe; SEM micrograph (**a**), EDX spectrum (**b**), FTIR spectrum (**c**), XRD pattern (**d**), N_2_ adsorption–desorption isotherm (**e**), and effect of pH on adsorption capacity of CHCFe (**f**). Conditions for (**f)**: initial pH of the solution = 2–10; mass of adsorbent = 0.05 mg; temperature = 25 °C;agitation time = 4 h; initial concentration of Congo red = 2 g L^–1^; volume of Congo red = 20 mL; and agitation speed = 160 rpm.

Scanning electron microscopy is a versatile method for visualisation of the microstructure morphologies of materials to obtain information such as the shape and size of the particles of the materials^27^. The surface of the CHCFe (Fig. 1a) is coarse and rough. There are a few non-uniform aggregates on the surface of the adsorbent. The surface is characterised with heterogeneous non-uniform particles, which dye molecules can adhere to. There are also holes on the surface that can encapsulate Congo red molecules^28^. The EDX spectrum (Fig. 1b) confirmed the presence of Fe, Si Al and Ca apart from the major elements, carbon and oxygen. The revelation of the elements is in agreement with the composition of the CHCFe.

Fourier transforms infrared spectroscopy is a technique that provides information on available active sites, which can bind or interact with pollutant molecules, on the surface of the adsorbent^29^ and this technique was used to visualise the active sites that present in CHCFe. The FTIR spectrum of CHCFe (Fig. 1c) reveals the following absorption bands: 3696, 3422, 1096, 1034, 909, 671, and 607–598 cm^–1^. These bands are correspondingly assigned to free –OH, –OH vibration (including hydrogen bonding), Si–C_6_H_5_ vibration, C–O stretch of alcohol or C–OH stretch or O–Si–O of silica or quartz, Al–OH deformation mode, –OH vibration mode, Fe–O vibration^3,21,27,30–33^.

The XRD diffraction pattern of CHCFe (Fig. 1d) and the quantification data (from XRD analysis) (Supplementary Fig. S2) reveal that there are four major compounds (alumina, silica, calcite or calcium carbonate and burnt ochre or iron oxide) in the composite. The composite is dominated by calcite (70%), followed by alumina (25%), burn ochre (4%), and silica (1%). The assignments of the peaks are: alumina (Al_2_O_3_) with 2θ of 25.584° (0 1 2), 43.363° (1 1 3), and 52.553° (0 2 4) (JCPDS card: 00-010-0173); silica or quartz (SiO_2_) with 2θ of 26.652° (1 0 1), and 45.809° (2 0 1) (JCPDS card: 00-033-1161); calcium carbonate (CaCO_3_) with 2θ of 29.406° (1 0 4), 31.418° (0 0 6), and 48.514° (1 1 6) (JCPDS card: 00-005-0586); and iron oxide (Fe_2_O_3_) with 2θ of 33.153° (1 0 4), 35.612° (1 1 0), and 40.855° (1 1 3) (JCPDS card: 00-033-0664). The CHFe has high degree of crystallinity.

The nitrogen adsorption–desorption isotherm of CHCFe (Fig. 1e) was used for evaluation of surface area and other parameters. The nitrogen adsorption–desorption data was subjected to Brunauer, Emmett and Teller (BET) and Barret, Joyner and Halenda (BJH) models. The parameters obtained from BET and BJH are BET surface area of 100.30 m^2^ g^–1^, pore volume of 0.099 cm^3^ g^–1^ and half pore width of 23.392 Å. The good surface area of the composite substantially enabled excellent adsorption of Congo red.

### Preliminary study and variation of pH on adsorption behaviour of CHCFe

The initial pH of the solution mostly has profound effects on adsorption capacities of adsorbents^5^. The effects of variation of pH on percentage adsorption of the dye by CHCFe (Fig. 1f) was investigated between pH 2 and 10, initial Congo red concentration of 2 g L^–1^, contact time of 4 h and 25 °C. The adsorbent adsorbed Congo red best at lower pH values. The percentage removal at pH 2 is 99.9 while that of pH 4 is 98.22. Hence, pH 2 is the optimal pH for the removal of Congo red by CHCFe. The result is in agreement with the previous report by Lafi et al.^10^, who reported pH 3 as optimum for removal of Congo red using activated coffee waste. Popoola et al.^5^ also reported optimum pH 3 for removal of Congo red using synthesised coal graphene. Removal of Congo red dye onto the adsorbents is usually influenced by pH because pH influences polarity of the surface of adsorbents as well as ionisation and ionic mobility of Congo red^13^. Congo red has a bipolar characteristic and can behave as anionic and cationic compound in solution depending on the pH of the medium^5^. This behaviour makes Congo red to change colour as pH is increased from acidic pH to alkaline pH. Removal of Congo red with anionic character is expected to increase as the initial pH of the solution decreases^10^. The value of *pH*_*pzc*_ of CHCFe is 6.97 (Supplementary Fig. S3) and this value signifies that the surface of the CHCFe is positively charged relative to the optimum pH, that is pH < pH_pzc_^5,7,25^.

In the preliminary experiments conducted at pH 2 (Supplementary Fig. S4), coconut husk, clay and coconut husk-clay (untreated) and coconut husk-clay-Fe composite were evaluated for their potentials to adsorb Congo red from aqueous solutions. The composite exhibited the best adsorption capacity than other adsorbents shown in the figure. Inclusion of iron compounds in the preparation of the composite could be responsible for surface activation, and hence increase the binding of Congo red molecules onto the surface of CHCFe.

### Kinetics of the adsorption process

**Figure 2:**
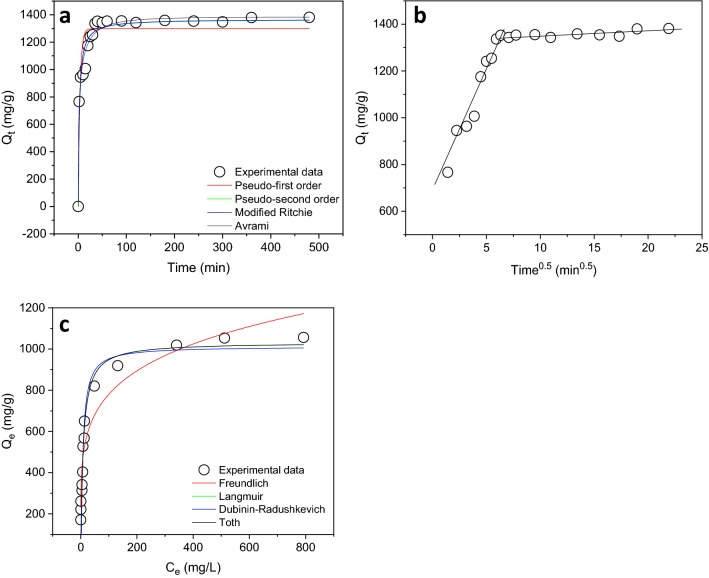
The curves of (**a** and **b**) kinetic modelling and (**c**) equilibrium modelling of data. Conditions: initial pH of the solution = 2; mass of adsorbent = 0.05 mg; temperature = 25 °C; agitation time = 0–480 min (kinetic study) and 1 h (equilibrium study); initial concentration of Congo red = 2 g L^–1^ (kinetic study) and 0.02–3 g L^–1^ (equilibrium study); volume of Congo red = 20 mL; and agitation speed = 160 rpm.

Amount of Congo red adsorbed by CHCFe is greatly time-dependent (Fig. 2a) at the beginning of the experiments. Amount of Congo red adsorbed increased progressively as the contact time increased. It means that there was a rapid removal of the dye molecules at the start of the adsorption process and this is because there were enough vacant binding sites on the surface of CHCFe at that moment. Adsorption of Congo red onto CHCFe was not time dependent after the equilibrium time of 40 min. Figure 2a also displays the modelling of time-dependent data to kinetic models. Pseudo-first order, pseudo-second order, modified Ritchie second order and Avrami fractional models were employed to elucidate the kinetic behaviour of the adsorption process, and the kinetic parameters (Table 1) were obtained from the models. To adjudge the best kinetic model, the values of *SD* and $R_{adj}^{2}$ were used. Avrami fractional model exhibited the lowest SD value and highest $R_{adj}^{2}$ value among the four kinetic models. This model presented the lowest difference between the values of experimental *q* (*Q*_*e(expt)*_) and calculated/theoretical *q* (*Q*_*e(calc)*_). Avrami model, which is an empirical model, is useful for explaining the behaviour of solid-solution interactions in adsorption systems^5,34,35^. The equilibrium adsorption value predicted by the model is 1383.3 mg g^–1^ at 25 °C while the value of Avrami rate constant (*k*_*AV*_) is 0.23737 min^–1^.

**Table 1:** Kinetic parameters of adsorption of Congo red by CHCFe.

Model	Parameters
*Q*_*e(exp.)*_ (mg g^–1^)	1355.3
**Pseudo-first order**	
*k*_*f*_ (min^–1^)	0.26518
*Q*_*e(cal.)*_ (mg g^–1^)	1299.1
*SD* (mg g^–1^)	119.08
$R_{adj}^{2}$	0.87510
**Pseudo-second order**	
*k*_*s*_ (g mg^–1^ min^–1^)	3.1100 × 10^–4^
*Q*_*e(cal.)*_ (mg g^–1^)	1366.9
*SD* (mg g^–1^)	70.539
$R_{adj}^{2}$	0.95617
**Modified Ritchie second order**	
*k*_*mR*_ (min^–1^)	0.41207
*Q*_*e(cal.)*_ (mg g^–1^)	1368.9
β (min^–1^)	1.0266
*SD* (mg g^–1^)	72.194
$R_{adj}^{2}$	0.95409
**Avrami fractional**	
*k*_*AV*_ (min^–1^)	0.23737
*Q*_*e(cal.)*_ (mg g^–1^)	1383.3
*n*_*AV*_	0.42685
*SD* (mg g^–1^)	52.794
$R_{adj}^{2}$	0.97545
**Intraparticle diffusion**	
*k*_*id,1*_ (mg g^–1^ min^–0.5^)	118.42
*C* (mg g^–1^)	617.42
$R_{adj}^{2}$	0.95681

Conditions: initial pH of the solution = 2; mass of adsorbent = 0.05 mg; temperature = 25 °C; agitation time = 0–480 min; initial concentration of Congo red = 2 g L^–1^; volume of Congo red = 20 mL; and agitation speed = 160 rpm.

The mechanism of adsorption process can be obtained from the analysis of kinetic data^36^. Therefore, the time-dependent data were equally subjected to intraparticle diffusion model (Fig. 2b) to ascertain the mechanism of the adsorption process. The intraparticle diffusion parameters (Table 1) were evaluated from the figure. If the kinetic data are subjected to intraparticle model and multilinear plots are obtained, it means that there are two or more adsorption stages that govern the adsorption process^37^. There are two adsorption stages for the removal of Congo red by CHCFe because there exist two linear sections in the intraparticle curve. The first linear adsorption stage (external mass transfer) is regarded as a fast-kinetic process and in this stage, there was a rapid migration of Congo red molecules to the surface of CHCFe. The second adsorption linear stage (intraparticle diffusion) is a stage that was attained after equilibrium. At this stage, there was a diffusion of the dye molecules through pores of CHCFe^38^. The intraparticle diffusion plot did not pass through the origin. This observation means that there were some levels of boundary layer control and the intraparticle diffusion was not the sole rate-limiting step that controlled the rate of adsorption^37^.

### Equilibrium/isotherm study

Adsorption isotherms describe the relationship between the concentration of substrate in solution and the amount of pollutant concentration (mg) adsorbed by a gram of adsorbent at a given temperature^2,39^. Hence, isotherm provides information on the interaction between the molecules of adsorbate in solution and the adsorbent surface^39^. Congo red removal by CHCFe at 25 °C (Fig. 2c) was concentration dependent, that is, Congo red uptake increased as the equilibrium concentration of dye increased. When the concentration of dye is low, the numbers of dye molecules available in the solution will be relatively low compared to the active sites on the surface of the adsorbent. At a high concentration of dye, more dye molecules will be present and these molecules will be more than what the active sites of adsorbent can take.

Equilibrium and isothermal data were subjected to four nonlinear models (Freundlich, Langmuir, Dubinin-Radushkevich and Toth) (Fig. 2c) with their parameters (Table 2) calculated from the models for all studies conducted at the temperature range 25–50 °C. The best model that explained the equilibrium adsorption of Congo red onto CHCFe is Langmuir. This model exhibited lowest *SD* values and highest $R_{adj}^{2}$ values than other models. The predicted Langmuir *Q*_*max*_ values obtained for Congo red adsorption onto CHCFe range between 888.85 and 1649.3 mg g^–1^. Hence, maximum uptake (1649.3 mg g^–1^) of Congo red took place at 50 °C.

**Table 2:** Equilibrium parameters of adsorption of Congo red by CHCFe.

Model/Temperature (°C)	25	30	35	40	50
**Langmuir**					
*Q*_*max*_ (mg g^–1^)	1032.5	1332.3	888.85	1378.5	1649.3
*K*_*L*_ (L mg^–1^)	0.11163	0.096700	0.072300	0.033420	0.013120
*SD* (mg g^–1^)	88.402	82.719	41.009	61.910	54.510
$R_{adj}^{2}$	0.92630	0.93454	0.98543	0.97495	0.92711
**Freundlich**					
*K*_*F*_ $(mg/g)(mg/L)^{{ - \frac{1}{{n_{F} }}}}$	315.87	89.562	266.46	118.25	135.56
*n*_*F*_	5.0918	2.3464	4.0525	2.6008	2.5185
*SD* (mg g^–1^)	90.611	106.27	43.656	76.497	147.39
$R_{adj}^{2}$	0.92257	0.91248	0.97545	0.95327	0.84644
**Dubinin-Radushkevich**					
*Q*_*max*_ (mg g^–1^)	1013.4	1152.9	823.83	1139.5	1498.2
*K*_*DR*_ (mol^2^ kJ^−2^)	1.1600 × 10^–3^	9.7800 × 10^–3^	7.4600 × 10^–4^	6.3700 × 10^–3^	7.9000 × 10^–3^
*SD* (mg g^–1^)	116.65	101.84	151.38	153.00	126.48
$R_{adj}^{2}$	0.87166	0.90079	0.70477	0.81308	0.88691
**Toth**					
*Q*_*max*_ (mg g^–1^)	1032.8	1348.1	814.19	1381.3	1632.7
*K*_*T*_ (L mg^−1^)	0.11161	0.0090800	0.26964	0.012250	0.013440
*n*_*T*_	2.3600 × 10^11^	3.8200 × 10^13^	1.9200 × 10^13^	3.9700 × 10^11^	1.0500 × 10^13^
*SD* (mg g^–1^)	92.331	86.845	139.15	113.95	140.34
$R_{adj}^{2}$	0.91960	0.92785	0.75055	0.89631	0.86078

Conditions: initial pH of the solution = 2; mass of adsorbent = 0.05 mg; temperature = 25–50 °C; agitation time = 1 h; initial concentration of Congo red = 0.02–3 g L^–1^; volume of Congo red = 20 mL; and agitation speed = 160 rpm.

An important parameter known as separation factor, *R*_*L*_, (Eq. 16) was evaluated from the Langmuir constant.

16 $$R_{L} = \frac{1}{{1 + K_{L} \cdot C_{o} }}$$

where *C*_o_ represents the initial concentration of Congo red in mg L^–1^. The values of this factor represent the behaviour of the isotherm as irreversible (*R*_*L*_ = 0) or linear (*R*_*L*_ = 1) or unfavourable (*R*_*L*_ > 1) or favourable (0 < *R*_*L*_ < 1). The values of *R*_*L*_ evaluated for adsorption Congo red by CHCFe range from 0.0059367 to 0.79214. The values of *R*_*L*_ (between 0 and 1) obtained indicate favourable adsorption of Congo red by CHCFe.

### Thermodynamic evaluation

Pieces of information on feasibility, spontaneity, nature (either endothermic or exothermic) and disorderliness of adsorption process are always obtained from temperature-dependent data. From the van’t Hoff plot (Supplementary Fig. S5), some of the thermodynamic parameters in Table 3 were evaluated. The values of standard Gibbs free energy (Δ*G*°) were calculated from the values of *K*_*L*_ (Langmuir constant; Langmuir being the best isotherm model). The values of Δ*G*° are in the range of –27.901 to –24.492 kJ mol^–1^. Negative values mean that the adsorption of Congo red onto CHCFe was generally spontaneous and feasible. The value of standard enthalpy change (Δ*H*°) is negative and this observation signifies that adsorption system was exothermic. In the same manner, the value of standard entropy change (Δ*S*°) is negative and this implies that there was a decrease in the randomness at Congo red solution–CHCFe interface during adsorption process^10,40^.

**Table 3:** Thermodynamic characteristics of the adsorption process.

Temp. (K)	*K*_*L*_ (L mol^–1^)	*ΔG°* (kJ mol^–1^)	*ΔH°* (kJ mol^–1^)	*ΔS°* (J mol^–1^ K^–1^)	$R_{adj}^{2}$	*RMSE*
298	77,769	− 27.901	− 72.239	− 146.86	0.93317	0.23001
303	67,368	− 28.008				
308	50,369	− 27.725				
313	23,283	− 26.167				
323	9140.2	− 24.492				

Conditions: initial pH of the solution = 2; mass of adsorbent = 0.05 mg; temperature = 25–50 °C; agitation time = 1 h; initial concentration of Congo red = 0.02–3 g L^–1^; volume of Congo red = 20 mL; and agitation speed = 160 rpm.

### Adsorption capacities of different adsorbents

The adsorptive performances of various adsorbents, including CHCFe, utilised for removal of Congo red were compared. Table 4 presents the comparison data. Among the twelve (12) adsorbents listed in the table, CHCFe outperformed all other adsorbents. Modification of the surface with Fe(II)/Fe(III) and good adsorbing feature of clay are some of the factors that are responsible for fantastic adsorption behaviour of CHCFe. The composite, CHCFe, could be utilised as an alternative and efficient adsorbent for Congo red removal from contaminated water.

**Table 4:** Comparison of CHCFe with other adsorbents for adsorption of Congo red.

Adsorbents	*Q*_*max*_ (mg g^–1^)	Temperature (°C)	Model	References
Ackee apple seed–bentonite composite	1439.9	25	Liu	^3^
Synthesised coal graphene	129.00	25	Liu	^5^
Pine bark	3.9200	25	Langmuir	^7^
Amino-functionalised silica gel	66.500	50	Langmuir	^9^
Activated carbon coffee waste	99.900	25	Langmuir	^10^
Clay–corn cob–FeCl_3_	1000.0	25	Langmuir	^11^
Periwinkle shell activated carbon	2.3585	50	Langmuir	^12^
Chemically modified rice husk char	2.0400	–	Langmuir	^13^
Water hyacinth bark carbon	14.367	50	Langmuir	^14^
Citric acid modified bentonite	384.00	30	Langmuir	^18^
Kaolinite supported CoFe_2_O_4_ nanoparticles	400.00	25	Langmuir	^21^
Coconut husk-clay-Fe composite	1649.3	50	Langmuir	*This study*

### Proposed mechanisms of adsorption

Adsorption could be described as a surface-based process in which molecules of an adsorbate (a substance in gaseous or liquid phase) are accumulated on the surface of an adsorbent’s material^41^. Mechanism of adsorption implies details of events that take place at each stage of adsorption or during entire adsorption process. There are different schools of thought on adsorption mechanisms, and adsorption mechanisms can be propositionally explained in several ways using different operating parameters. The mechanisms of Congo red adsorption onto CHCFe will be discussed by using (1) type of functional groups present in CHCFe, (2) nature of the isotherm curve, (3) initial pH of the Congo red solution, (4) the linearity of intraparticle diffusion curve, and (5) thermodynamic data.

As discussed earlier, removal of Congo by CHCFe followed Langmuir equilibrium model. This model assumes that (1) adsorbate molecules are adsorbed on the binding or active sites located on the adsorbent’s surface, (2) all the adsorption sites are energetically identical, (3) the binding of adsorbate on the adsorbent’s surface is monolayer adsorption, (4) each active site can only bind a molecule of an adsorbate at a time, and (5) there is no interaction among the adsorbed molecules on adsorbent surface^42–44^. On the basis of IUPAC classification of adsorption isotherm, the isotherm of CHCFe (Fig. 2c) followed Type L isotherm with a long well-defined plateau^41,45^. In this isotherm, it is assumed that adsorption of adsorbate occurred on a microporous adsorbent in which the adsorbent’s surface is covered with a monolayer of adsorbate^43^.

Congo red, an anionic/acidic dye, has sulphonic group (–SO_3_–) in its structure^10^. At a low pH, Congo red dye will dissociate to a polar group (R–SO_3_–), hence, Congo red will be favourably adsorbed at low pH since the surface of CHCFe is positively charged relative to the dye molecules^10,13^. The opposing charges on the surface of CHCFe and dye molecules enhanced electrostatic interactions between CHCFe surface and R–SO_3_– of dye molecules^10^. The adsorbent surface that was positively charged favoured the binding and sequestration of anionic Congo red from aqueous solutions.

**Figure 3:**
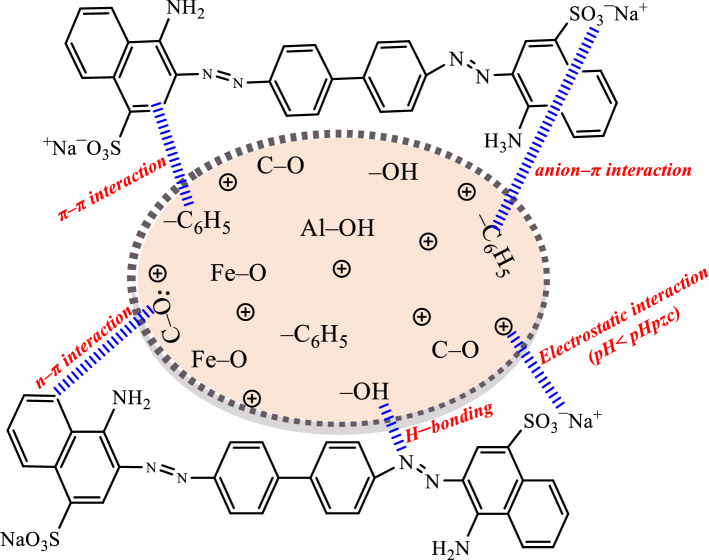
Proposed mechanisms of adsorption of Congo red by CHCFe.

The FTIR data of CHCFe showed that the major functional groups present in the adsorbent are –OH, –C_6_H_5_, C–O, O–Si–O, Al–OH and Fe–O. Interactions of adsorbent with adsorbate, which will eventually lead to removal of adsorbate from aqueous solutions, include hydrogen bonding, π–π interaction, n–π interaction, anion–π interaction, electrostatic interaction, dispersion interaction, and hydrophobic interaction^8,30,46^. Some of the existing interactions between Congo red and CHCFe include hydrogen bonding, n–π, π–π, anion–π, and electrostatic (Fig. 3).

Weber and Morris^47^ proposed intraparticle diffusion model for explanation of the adsorption process if the diffusion of adsorbate to the porous adsorbent is the rate-limiting step. In the present study, adsorption of Congo red onto CHCFe is biphasic, that is, bilinear curve was obtained. This scenario confirmed that adsorption process occurred through a fast external mass transfer of adsorbate followed by a slow intraparticle diffusion of adsorbate molecules into the inner pores of CHCFe. There exist variations in mass transfer of Congo red at the initial and final stages, hence, the linear segments of the plot did not pass through the origin^25^. In addition to this, diffusion was not the only mechanism responsible for Congo red adsorption onto CHCFe and that the adsorption process was governed by either pore or film diffusion^37,48^. The value of *C* (617.42 mg g^–1^) obtained for CHCFe-Congo red interaction is high. This value is higher than the values reported for a few studies for adsorption of Congo red^3,5,10,11^. When the value of C is high, it means that the effect of the boundary layer is pronounced^43^.

Molecules of adsorbate can be adsorbed onto the surface of adsorbent via physisorption (physical adsorption) or chemisorption (chemical adsorption) depending of the type of interaction between the adsorbent material and adsorbate. Physisorption is always exothermic and occurs at high relative pressures, physisorption process mainly takes place as a multilayer. A physisorption process will possess adsorption energy < 40 kJ mol^–1^ but a chemisorption process possess will exhibit energy > 40 kJ mol^–1^^46,49,50^. The value of adsorption energy (enthalpy change) for removal of Congo red by CHCFe is − 72.239 kJ mol^–1^, and this signifies that physisorption process predominates during adsorption process.

## Conclusion

This research prepared an efficient adsorbent material from raw clay, coconut husk, and inorganic compounds and the adsorbent (CHCFe) was utilised for removal of Congo red from aqueous solution in a process that is feasible, spontaneous and exothermic. The data from FTIR, SEM–EDX, N_2_ adsorption–desorption, XRD, and pHpzc of CHCFe correspondingly revealed that the adsorbent possessed suitable adsorptive functional groups, heterogeneous surface, good surface area, four major compounds, and positive surface. The rate of removal of Congo red by CHCFe was a fast process and followed Avrami fractional model. The adsorption process occurred in two phases: mass migration of Congo red molecules onto the CHCFe and intraparticle diffusion of the dye molecules through the inner pores. The CHCFe-Congo red system was spontaneous, feasible and exothermic. The value Δ*S*° for the adsorption process is negative—an indication that a decrease in the randomness at Congo red solution–CHCFe interface manifested during adsorption process. The maximum Congo red uptake by CHCFe as suggested by Langmuir model is 1649.3 mg g^–1^ and this occurred at 50 °C. The adsorbent (CHCFe) could be deployed for treatment of wastewater contaminated with dyes, especially Congo red.

## Supplementary Information

Supplementary Information.

## Data Availability

All data generated or analysed during this research are included in this published article and its supplementary information files.
