# Supplementary material for: Coconut husk-raw clay-Fe composite: preparation, characteristics and mechanisms of Congo red adsorption
Source: Sci Rep. 2022 Aug 23;12:14370. doi: 10.1038/s41598-022-18763-y (PMC9399237; doi:10.1038/s41598-022-18763-y)
Supplement: Supplementary file 1 — Supplementary Information. [file 41598_2022_18763_MOESM1_ESM.pdf]

Supplementary Information

Coconut husk-raw clay-Fe composite: Preparation, characteristics and mechanisms of Congo red adsorption

Matthew Ayorinde Adebayo[1,*], Jamiu Mosebolatan Jabar[1], Justinah Solayide Amoko[2], Elijah Ojo Openiyi[1,3] and Olamide Oladimeji Shodiya[1]

[1]Department of Chemistry, The Federal University of Technology, Akure, Ondo State, Nigeria

[2]Department of Chemistry, Adeyemi College of Education, Ondo, Ondo State, Nigeria

[3]Interdisciplinary Ecological Sciences and Engineering, Purdue University, West Lafayette, IN 47906, USA

*All correspondence to: adebayoma@futa.edu.ng (M.A. Adebayo)

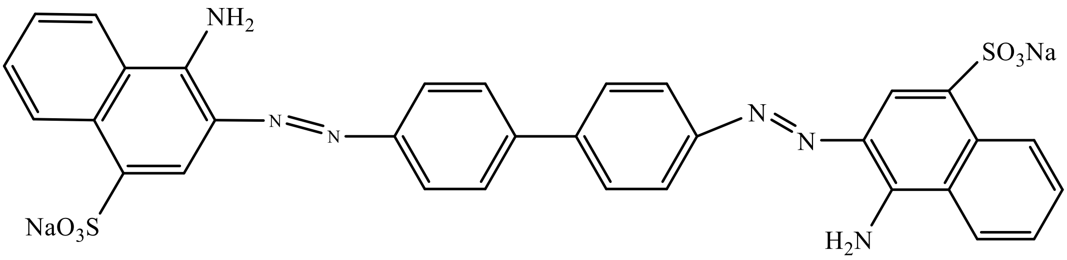

Supplementary Figure S1. The chemical structure of Congo red dye

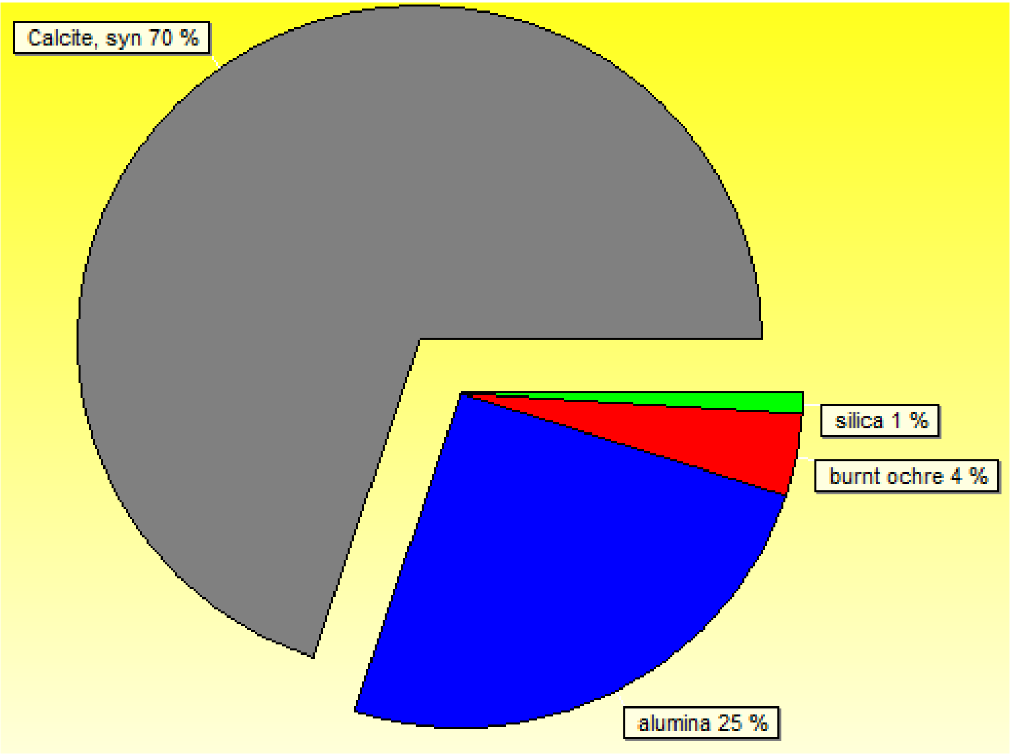

Supplementary Figure S2. Composition of CHCFe obtained *via* XRD analysis

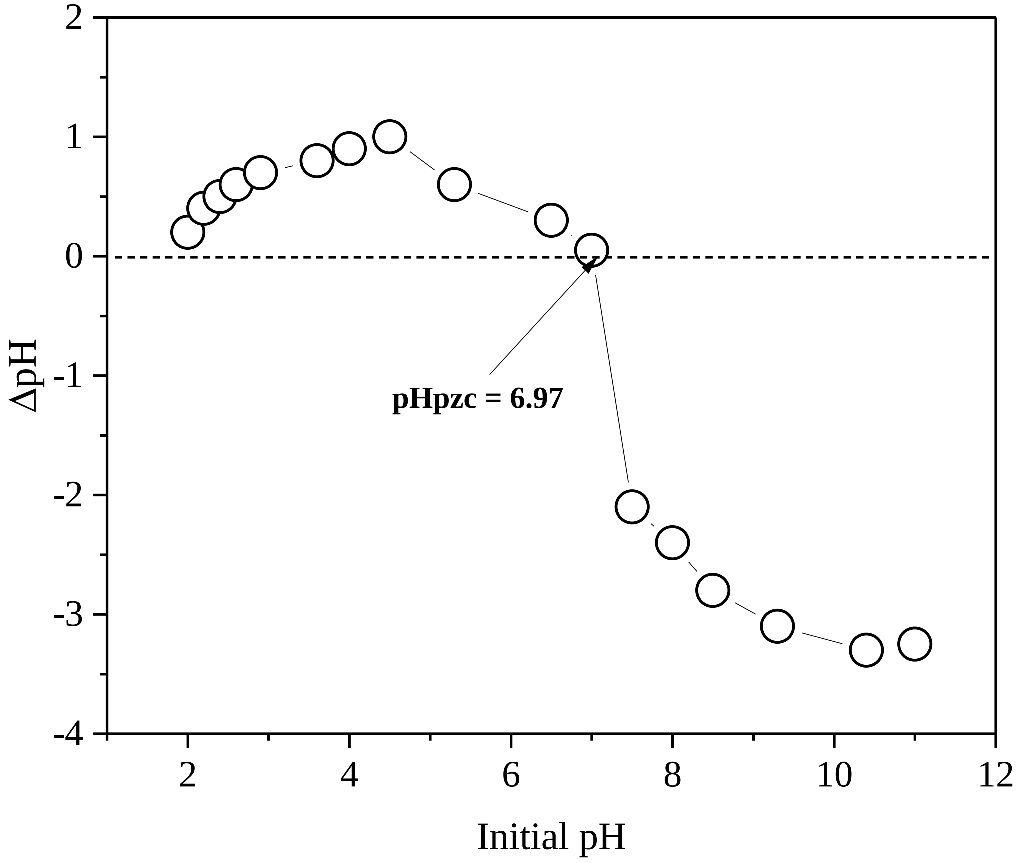

Supplementary Figure S3. *pHpzc* of CHCFe

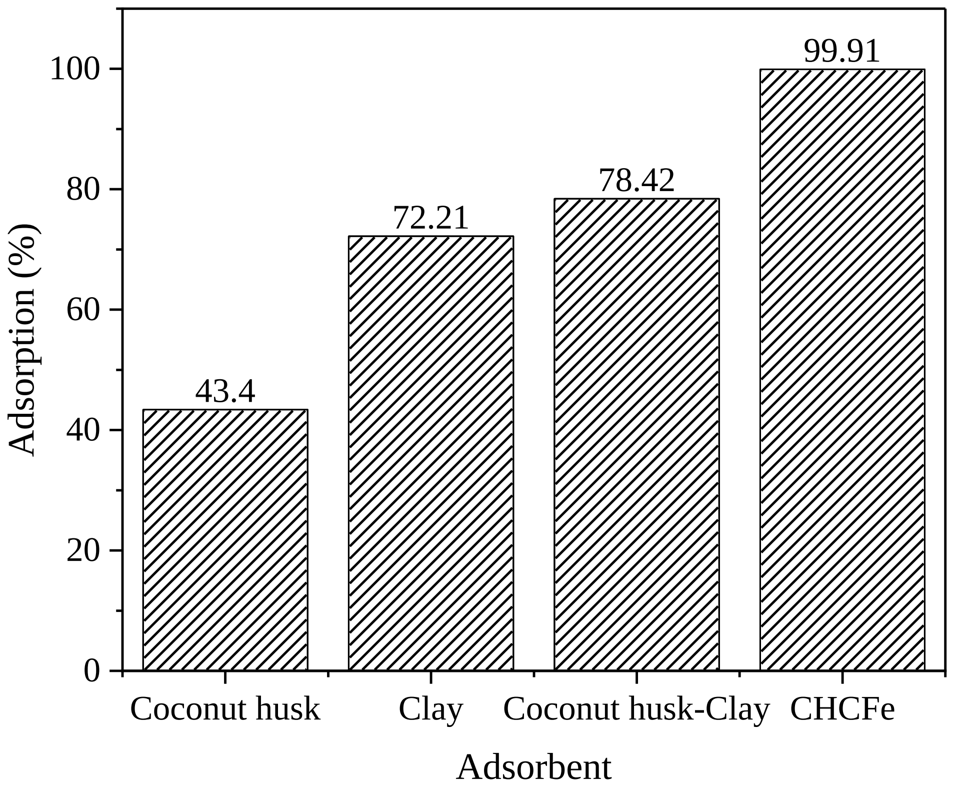

Supplementary Figure S4. Preliminary adsorption capacities at pH 2

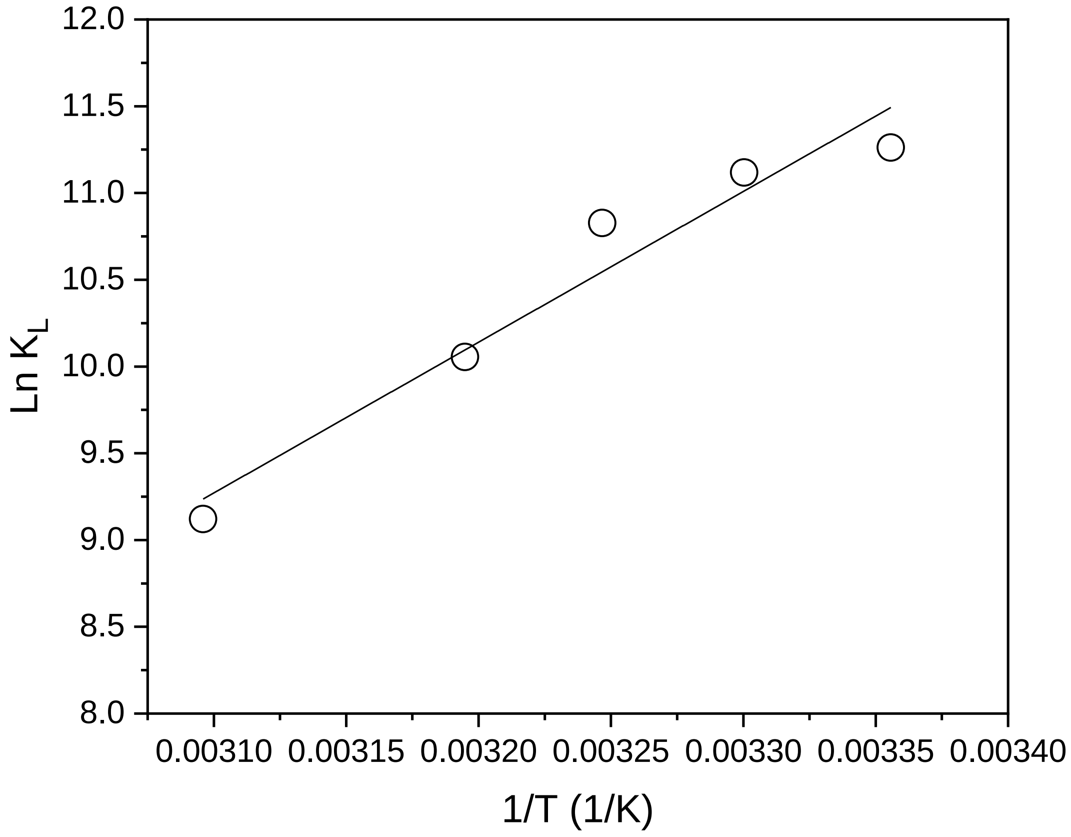

Supplementary Figure S5. van’t Hoff plot for the removal of Congo red by CHCFe
